# Supplementary material for: Characterization of Salix nigra floral insect community and activity of three native Andrena bees
Source: Ecol Evol. 2021 Mar 18;11(9):4688–700. doi: 10.1002/ece3.7369 (PMC8093708; doi:10.1002/ece3.7369)

**Table S1.** List of characterized floral VOCs. VOCs with Vector Max R > 0.4000 (bolded) were selected for Pearson correlation with individual abundant floral visitors.

| Volatile | Type | Vector Max R |
| --- | --- | --- |
| **Trans-3-Pinanone** | **Monoterpene** | **0.5563** |
| **Cis-b-Terpineol_2** | **Monoterpene** | **0.4254** |
| 5-9-Undecadien | Monoterpene | 0.3557 |
| Cis-b-Terpineol_1 | Monoterpene | 0.3554 |
| Sabinene | Monoterpene | 0.2732 |
| Dimethylnona | Monoterpene | 0.2373 |
| Cis-ocimene | Monoterpene | 0.224 |
| Terpinolene | Monoterpene | 0.2097 |
| Eucalyptol | Monoterpene | 0.1983 |
| Trans-ocimene | Monoterpene | 0.1713 |
| Myrcene | Monoterpene | 0.1571 |
| Limonene | Monoterpene | 0.0783 |
| b-pinene | Monoterpene | 0.0662 |
| g-terpinene | Monoterpene | 0.0611 |
| a-pinene | Monoterpene | 0.0492 |
| **Hexenyl acetate** | **Other** | **0.6436** |
| **Octanal** | **Other** | **0.5951** |
| **Octen-2-ol** | **Other** | **0.5815** |
| **Acetophenone** | **Other** | **0.4446** |
| **Ethyl-1-hexanol** | **Other** | **0.4084** |
| Hexenyl-benzoate | Other | 0.3015 |
| Me-salicylate | Other | 0.2864 |
| Methylpentanol | Other | 0.2727 |
| Benzenealdehyde | Other | 0.2384 |
| Benzaldehyde | Other | 0.2324 |
| Tridecane | Other | 0.2281 |
| Component_40 | Other | 0.2006 |
| Benzyl-cyanide | Other | 0.1454 |
| Me-5-Hepten-2-one | Other | 0.1259 |
| p-cymene | Other | 0.0795 |
| Component-23 | Other | 0.0488 |
| Hexen-1-ol-AC | Other | 0.0396 |
| Benzeneethanol | Other | 0.0172 |
| **Germacrene-D** | **Sesquiterpene** | **0.4754** |
| Cubebol | Sesquiterpene | 0.3543 |
| Caryophyllene | Sesquiterpene | 0.2539 |
| E-E-a-Farnesene | Sesquiterpene | 0.2482 |
| b-Bourbonene | Sesquiterpene | 0.2331 |
| Z-E-a-Farnesene | Sesquiterpene | 0.2057 |
| Humulene | Sesquiterpene | 0.1844 |
| Copaene | Sesquiterpene | 0.1544 |

**Table S2.** Environmental fit results for 2019 floral community NMDS analysis. Bolded p-values (<0.05) indicate a significant correlation of the independent variable with the NMDS configuration.

| Independent variable | Vector Max R | p-value |
| --- | --- | --- |
| Julian date | 0.3036 | **0.001** |
| Temperature | 0.0582 | 0.241 |
| Military time | 0.0447 | 0.308 |

**Table S3.** Nested ANCOVA model results for 2019 analysis of individual native bee abundances. Bolded p-values (<0.05) indicate significant model effects.

y ~ Sex + Location + Tree(Sex)&Random + Julian date

| Dependent variable: *Andrena nigrae* | | | | | |
| --- | --- | --- | --- | --- | --- |
| Source | Degrees of freedom | Sum of squares | Mean square | F-Ratio | p-value |
| Model | 13 | 43.48185 | 3.34476 | 2.4947 | **0.0134** |
| Error | 40 | 53.62887 | 1.34072 |  |  |
| C. Total | 53 | 97.11072 |  |  |  |
| Dependent variable: *Andrena macoupinense* | | | | | |
| Source | Degrees of freedom | Sum of squares | Mean square | F-Ratio | p-value |
| Model | 13 | 5.604838 | 0.431141 | 3.9674 | **0.0004** |
| Error | 40 | 4.34681 | 0.10867 |  |  |
| C. Total | 53 | 9.951648 |  |  |  |
| Dependent variable: *Andrena morrisonella* | | | | | |
| Source | Degrees of freedom | Sum of squares | Mean square | F-Ratio | p-value |
| Model | 13 | 4.347317 | 0.334409 | 2.9616 | **0.0042** |
| Error | 40 | 4.516573 | 0.112914 |  |  |
| C. Total | 53 | 8.86389 |  |  |  |
| Dependent variable: *Lasioglossum coeruleum* | | | | | |
| Source | Degrees of freedom | Sum of squares | Mean square | F-Ratio | p-value |
| Model | 13 | 3.101719 | 0.238594 | 2.2816 | **0.023** |
| Error | 40 | 4.182974 | 0.104574 |  |  |
| C. Total | 53 | 7.284693 |  |  |  |

**Table S4.** Nested ANCOVA model results for 2019 analysis of calculated community metrics. Bolded p-values (<0.05) indicate significant model effects.

y ~ Sex + Location + Tree(Sex)&Random + Julian date

| Dependent variable: Species richness | | | | | |
| --- | --- | --- | --- | --- | --- |
| Source | Degrees of freedom | Sum of squares | Mean square | F-Ratio | p-value |
| Model | 13 | 307.5722 | 23.6594 | 3.3871 | **0.0015** |
| Error | 40 | 279.4093 | 6.9852 |  |  |
| C. Total | 53 | 586.9815 |  |  |  |
| Dependent variable: Shannon-Weaver diversity | | | | | |
| Source | Degrees of freedom | Sum of squares | Mean square | F-Ratio | p-value |
| Model | 13 | 9.700905 | 0.746223 | 5.2588 | **<0.0001** |
| Error | 40 | 5.675971 | 0.141899 |  |  |
| C. Total | 53 | 15.37688 |  |  |  |

**Table S5.** Environmental fit results for floral community NMDS analysis across years (2017-2019). Bolded p-values (<0.05) indicate a significant correlation of the independent variable with the NMDS configuration.

| Independent variable | Vector Max R | p-value |
| --- | --- | --- |
| Year | 0.0802 | **0.047** |
| Julian date | 0.0815 | **0.046** |
| Temperature | 0.0491 | 0.18 |

**Table S6.** Nested ANCOVA model results for year analysis. Bolded p-values (<0.05) indicate significant model effects.

y ~ Sex + Year + Tree(Sex)&Random + Julian date

| Dependent variable: *Andrena macoupinense* & *Andrena morrisonella* | | | | | |
| --- | --- | --- | --- | --- | --- |
| Source | Degrees of freedom | Sum of squares | Mean square | F-Ratio | p-value |
| Model | 8 | 27.17312 | 3.39664 | 1.6702 | 0.1218 |
| Error | 68 | 138.2903 | 2.03368 |  |  |
| C. Total | 76 | 165.4635 |  |  |  |
| Dependent variable: *Andrena nigrae* | | | | | |
| Source | Degrees of freedom | Sum of squares | Mean square | F-Ratio | p-value |
| Model | 8 | 46.10628 | 5.76328 | 4.9103 | **<0.0001** |
| Error | 68 | 79.81222 | 1.17371 |  |  |
| C. Total | 76 | 125.9185 |  |  |  |
| Dependent variable: Species richness | | | | | |
| Source | Degrees of freedom | Sum of squares | Mean square | F-Ratio | p-value |
| Model | 8 | 82.47375 | 10.3092 | 4.8422 | **<0.0001** |
| Error | 68 | 144.773 | 2.129 |  |  |
| C. Total | 76 | 227.2468 |  |  |  |
| Dependent variable: Shannon-Weaver diversity | | | | | |
| Source | Degrees of freedom | Sum of squares | Mean square | F-Ratio | p-value |
| Model | 8 | 5.76738 | 0.720923 | 7.3942 | **<0.0001** |
| Error | 68 | 6.62987 | 0.097498 |  |  |
| C. Total | 76 | 12.39725 |  |  |  |

**Figure S1.** Tree canopy pan-trap design. Camouflaged buckets were added to hang below river traps to add weight, preventing winds from jostling traps.

**Figure S2.** Total concentration of catkin metabolites, leaf metabolites and catkin VOCs for male and female trees. Letters to the left of boxes indicate significantly different means (p-value < 0.05) determined by one-way ANOVA test.

**Figure S3.** Total concentration averages of monoterpenes and sesquiterpenes VOCs for male and female trees. Letters to the left of boxes indicate significantly different means (p-value < 0.05) determined by one-way ANOVA test.

**Figure S4.** Correlation of *A. nigrae* with VOC compounds.

**Figure S5.** Average values of species richness and Shannon-Weaver diversity for female and male trees from year analysis model. Letters to the left of boxes indicate significantly different means as determined by a Tukey’s HSD (p-value < 0.05) for each separate nested ANCOVA model.

**Figure S6.** Photographs of size and color differences characteristic of a) male and b) female flowers in full bloom at field site.

**Figure S1.**


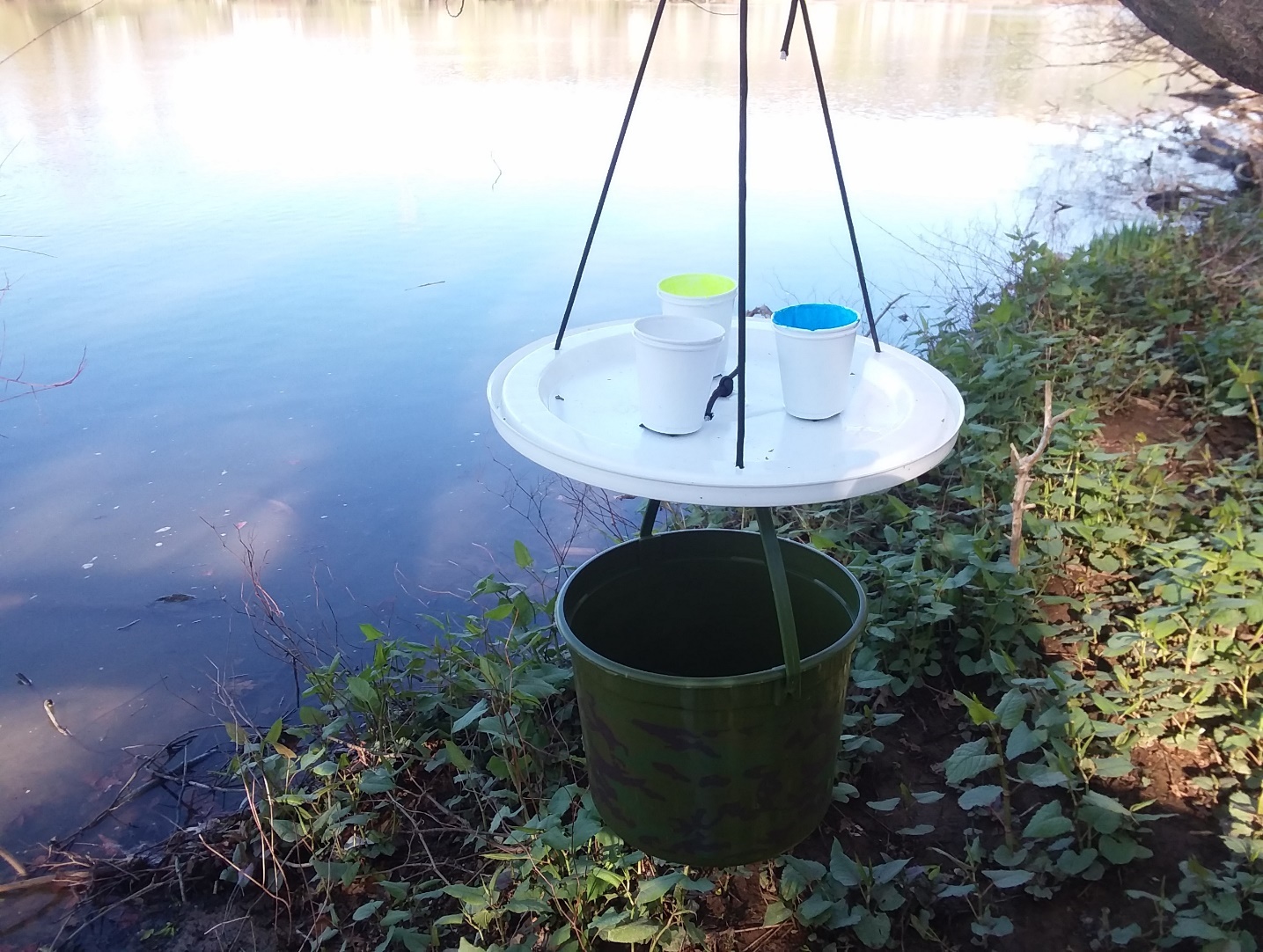


**Figure S2.**


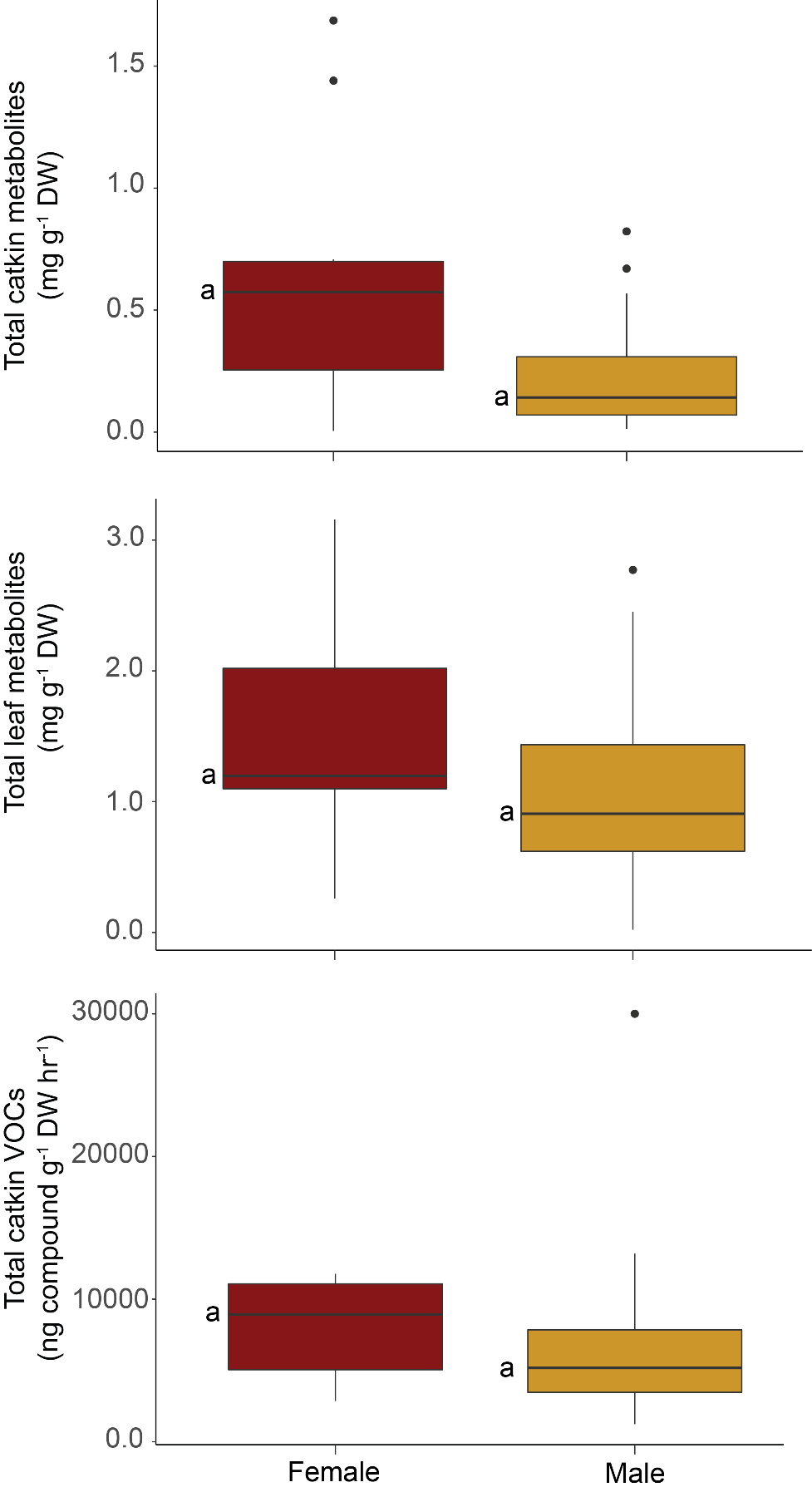


**Figure S3.**
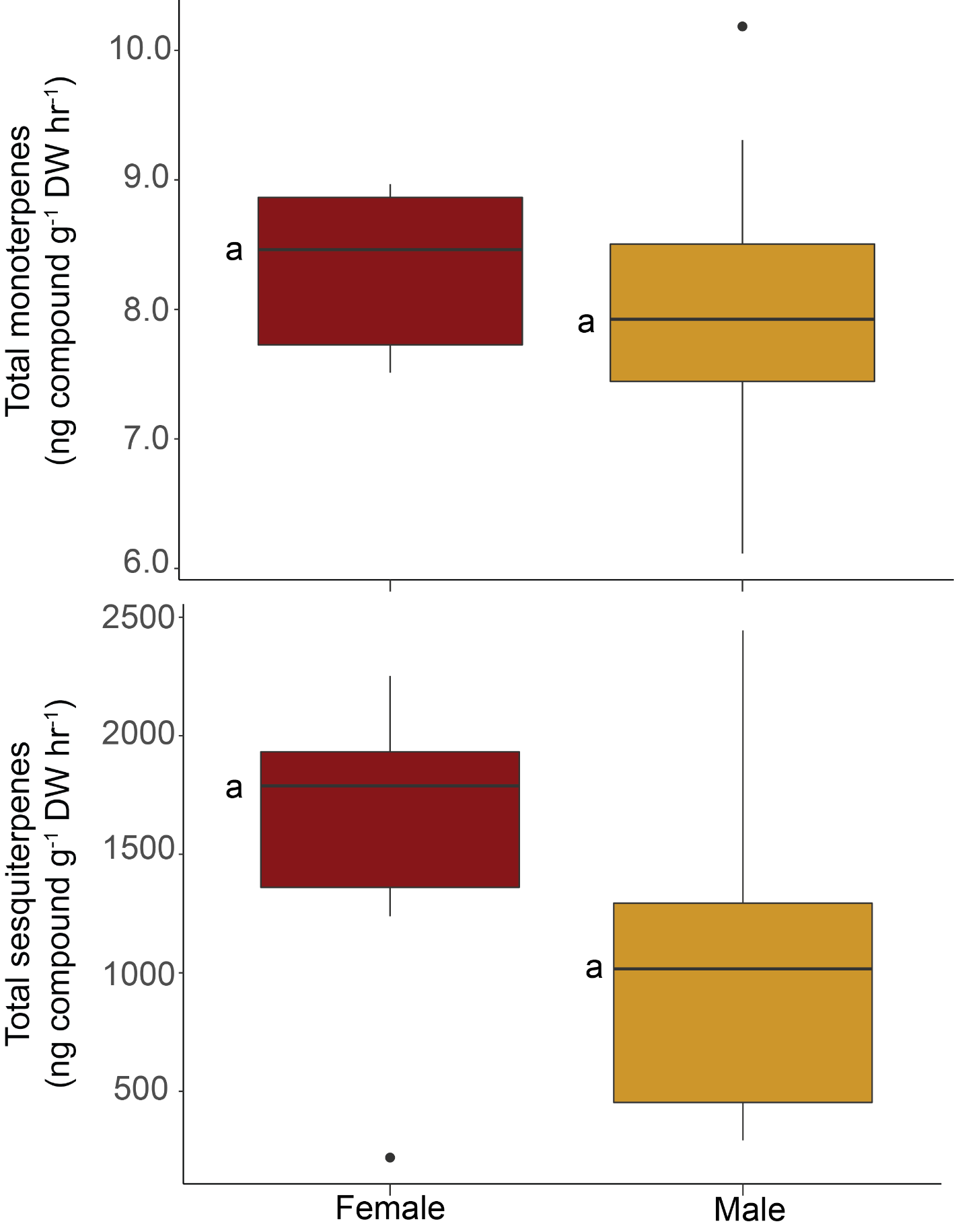


**Figure S4.**


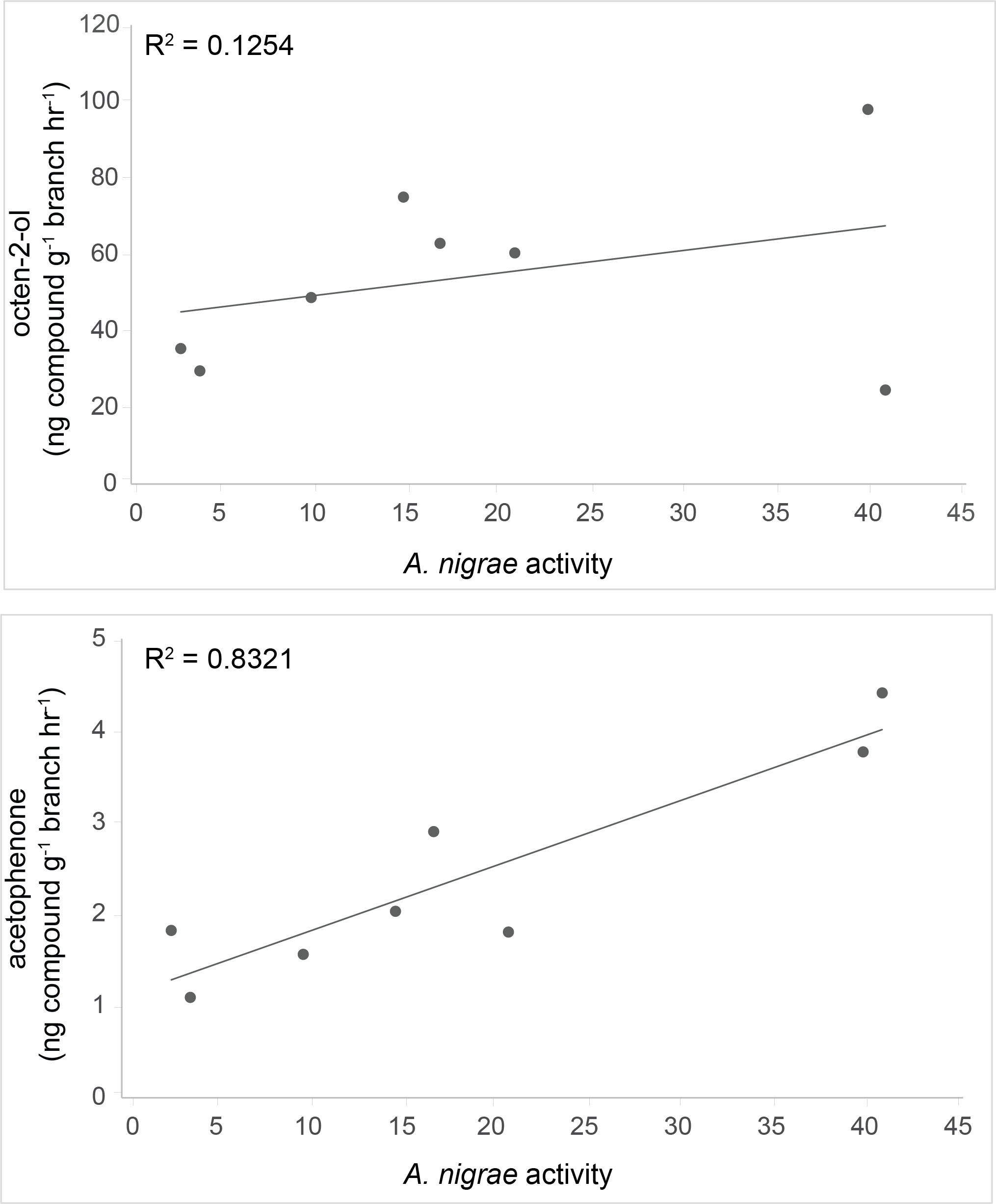


**Figure S5.**
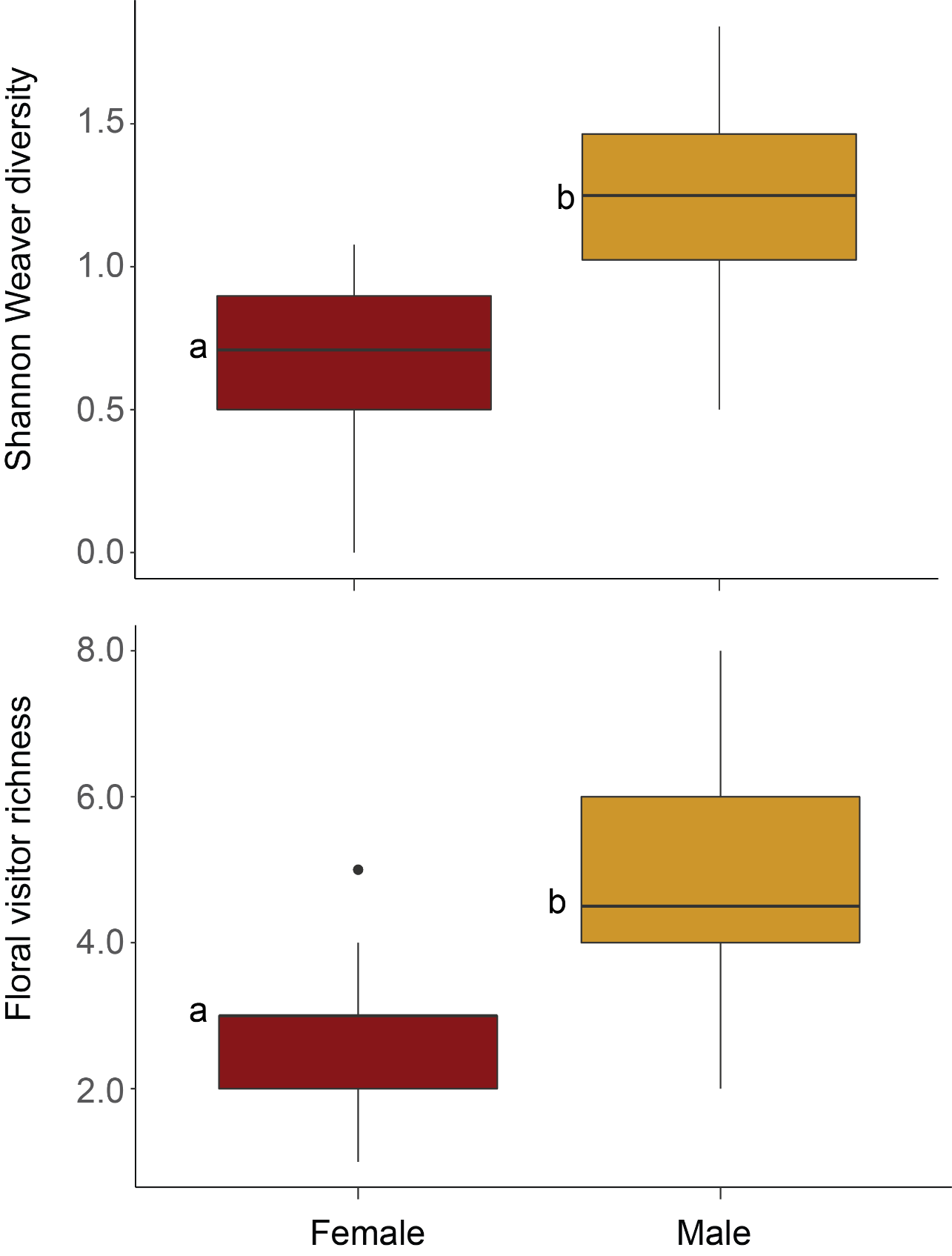


**Figure S6.**


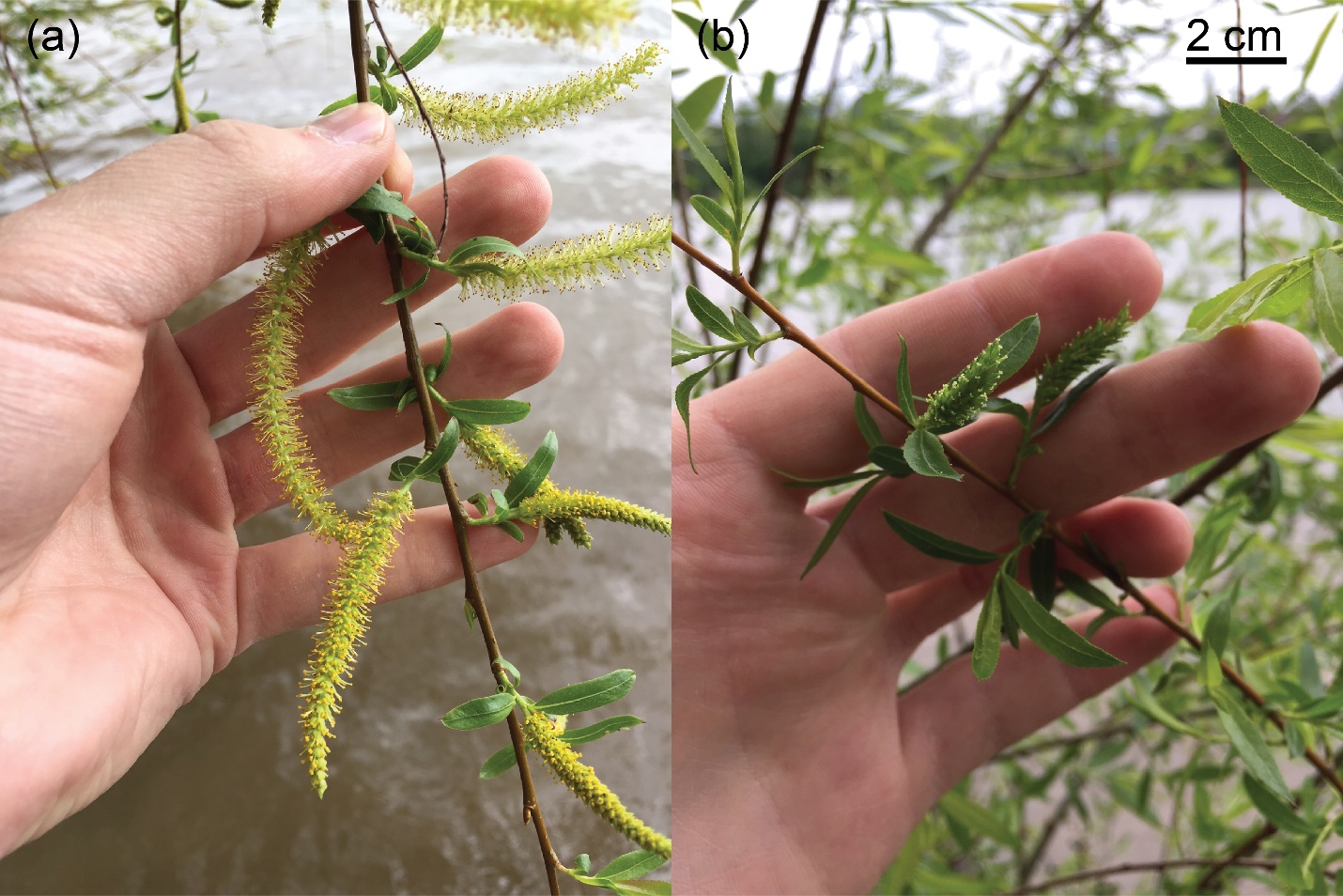

Supplement: Supplementary file 1 — Supplementary Material [file ECE3-11-4688-s001.docx]
